# Standardization in Transmission Spectrophotometry in the Visible and Ultraviolet Spectral Regions*

**DOI:** 10.6028/jres.080A.061

**Published:** 1976-08-01

**Authors:** A. R. Robertson

**Affiliations:** Division of Physics, National Research Council, Ottawa, Ontario, Canada K1A OR6

**Keywords:** Errors in spectrophotometry, photometric scale, slit width, spectral transmission, spectrophotometer standards, stray light, wavelength scale

## Abstract

In an instrument as complex as a spectrophotometer there are many potential sources of error. Because of this it is useful to have available standard materials whose spectral transmittances are known accurately. Periodic measurement of such standards provides a useful indication of whether a spectrophotometer is producing accurate results.

If the spectral transmittance functions of these standards are chosen suitably, the measurements can provide diagnostic information to indicate what type of error is occurring. Among the factors that most often lead to errors in spectrophotometry are the slit-width, the wavelength scale, the photometric scale, and stray radiation. Suitable material standards can provide indications of the occurrence of these errors. However it is sometimes difficult to identify a particular error since often several errors will occur at the same time.

Several sets of standards for testing spectrophotometers are available or can be constructed easily. Most of these are glass filters, but interference filters, perforated screens, and rotating sectors are also used. Liquid filters have some advantages, especially in the ultraviolet where glass filters absorb too strongly to be useful. However difficulties in preparing and handling liquid filters can introduce uncertainties.

It is important that standard materials are insensitive to environmental conditions (such as temperature) and that they are stable over a long period of time. Unfortunately, many of the materials with the most suitable spectral characteristics are least suitable in these respects, and it would be very useful if new and better materials could be developed.

## I. Introduction

A spectrophotometer is an extremely complex instrument and there are many potential sources of error. Tests are available that will detect the presence of each type of error but they are usually rather inconvenient to use in practice. It is often more convenient to have available a set of material standards whose spectral transmittances are known accurately. If the spectral transmittances are chosen carefully, measurement of them can provide a useful check on the performance of the instrument. There is no need to modify or make any additions to the instrument because the tests can be made with the instrument in the mode in which samples are normally measured.

In general, departure of the measured transmittance from the true transmittance will indicate the presence of an error, but will not indicate the type of error (photometric, wavelength, silt-width, stray radiation, etc.). However, if the spectral transmittances of the standards are chosen appropriately, it is sometimes possible to use the measurements to diagnose what type of error is occurring. The spectral transmittances must be chosen so that the error in question will have the maximum effect while other errors have very little or no effect. A problem is that two different errors will sometimes have the same or similar effects on the measured transmittances making it difficult to interpret the measurements, but nevertheless, departure of the measured transmittances from the true transmittances will always be an indication of problems even when diagnostic information cannot be derived.

If the standards are calibrated in one instrument and then used in another instrument of different design the situation may be very complex, and it will be quite difficult to determine exactly what is causing differences in the results from the two instruments, especially when more than one factor is involved. Standards are often much more valuable when they are used to detect changes in the performance of a single instrument. Periodic measurement of standard materials will give an early warning of problems that may be developing from dirty optical components, from wear of mechanical components, from aging of the source or detector or from any other cause. Standards are also useful in detecting and correcting faulty calibration or faulty experimental technique.

As well as having the desired spectral transmittance curves, materials to be used as standards must have certain other properties. They must be stable with respect to time, temperature, humidity and other environmental parameters; they must be uniform, nonscattering, nonfluorescent, and nonpolarizing; and they must be easy to handle.

**Figure 1:**
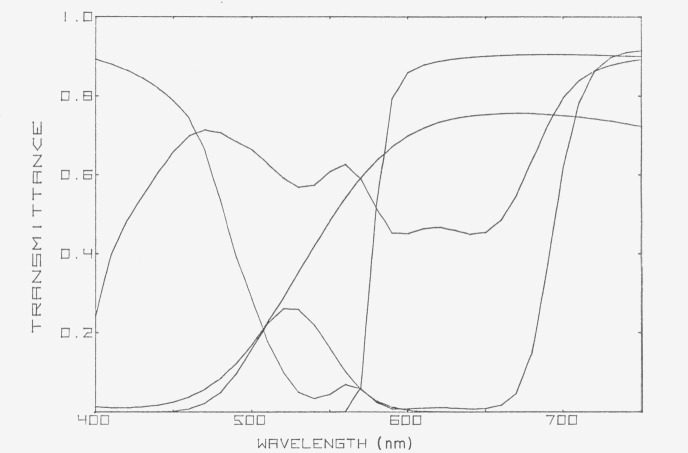
Spectral transmittances of the NBS 2100 series of five colored-glass filters for checking the performance of spectrophotometers [1].

An example of a set of standards that is very useful in detecting and diagnosing errors in the visible spectral region is the 2100 series of five colored-glass filters issued by the National Bureau of Standards [1].^1^ The spectral transmittances of these filters are shown in figure 1.

## II. Use of Standards to Determine Different Types of Error

In this section some of the many types of error that can occur in spectrophotometry are discussed. Material standards that can indicate the presence of each type of error are described. Errors that can occur with special types of samples such as samples that fluoresce or that change the state of polarization of the measuring beam are not discussed.

### A. Photometric Errors

Photometric errors can conveniently be divided into two types: errors of the endpoints of the photometic scale (0% and 100% transmittance) and failure of readings taken from the photometric scale to be linearly related to the amount of radiation striking the photodetector. Endpoint errors can be detected easily by measurements with an opaque sample and with an empty sample compartment, but linearity is more difficult to check. A survey of methods of measuring photometric linearity has been given by Sanders [2]. The most fundamental method is the flux addition or superposition method. The simplest form of this is the double- aperture method described by Clarke [3] and by Mielenz and Eckerle [4]. The method requires special equipment and there are several systematic errors that may occur, so it is more convenient in routine work to use standards of known transmittance. To reduce or eliminate the effect of other errors, these standards should have a transmittance that does not vary with wavelength, but this is difficult to achieve in practice.

Filters made of metal screens with a large number of small holes have been suggested but there are problems with diffraction effects and with dirt accumulating in the holes [5]. In addition the position of the screens in the measuring beam is critical, especially if the cross-section of the beam is small or nonuniform. Rotating sectors are sometimes used but they may be too large to fit into the sample compartment and, in any case, their use depends on the assumption that the photo-electronic system obeys Talbot’s law, an assumption that may not always be valid. Interference filters can be made spectrally neutral, but they reflect rather than absorb the untransmitted radiation so they may introduce stray radiation errors. In addition, pin-holes and other nonuniformities in these filters can cause errors.

**Figure 2:**
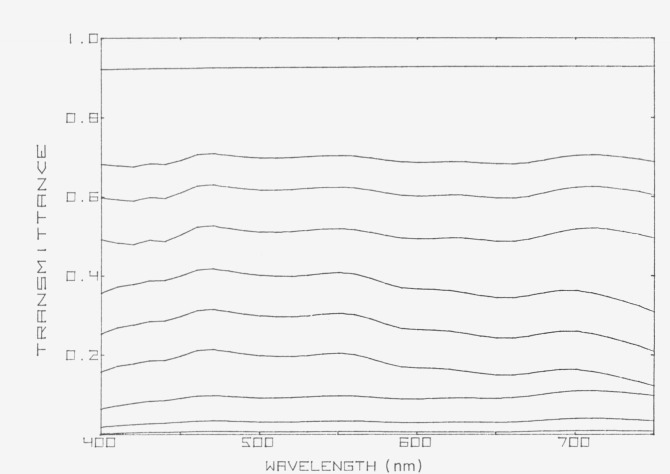
Spectral transmittances of ten neutral glass filters that have been used to check photometric scales.

Colored glass filters with an approximately neutral spectrum are probably the most satisfactory materials for checking photometric scales, but they too have some disadvantages. The spectral transmittances of a set of neutral glass filters that has been used at the National Research Council are shown in figure 2. A calibrated set of three similar filters is available from the National Bureau of Standards [7]. Such filters can be used with the least uncertainty to check for changes in the photometric linearity of a single instrument. When they are used to compare two different instruments, the uncertainties are higher because geometrical differences between the two instruments may lead to different effective pathlengths through the filters. A disadvantage of these materials is that they are not perfectly neutral, so that wavelength and bandwidth errors may affect their transmittance, unless only the flat parts of the spectral transmittance curve are used, but even then stray radiation errors may influence the results. Another disadvantage is that the filters are not always stable over long periods of time[6]. Surface films of silicon oxide may form[7, 8] and there may be some leaching of metal oxides from the surface caused by the action of the atmosphere or by cleaning agents [3].

It must be noted that colored glasses can only be used to check a photometric scale if the transmittances of the glasses have been established in advance with an instrument whose linearity has been checked by a fundamental method such as the double aperture method. There is no way that they can b<3 used on their own to establish photometric linearity [6]. It is sometimes assumed fallaciously that if the transmittances of two thicknesses of the same filter material (or two thicknesses or two concentrations of a liquid) obey the Lambert-Beer law (after suitable correction for surface reflections), then the instrument is linear. The justification of this fallacy is made as follows. Let the true spectral internal transmittances of two filters of the same material be *T*_1_(λ) and *T*_2_(λ), the thicknesses *d*_1_(λ) and *d*_2_(λ), and the spectral absorptivity *a*(λ), then

$$T_{1}(\lambda )=10^{-d_{1}a(\lambda )}$$

and

$$T_{2}(\lambda )=10^{-d_{2}a(\lambda )}$$

Thus the ratio of the spectral absorbances *A*_1_(λ) and *A*_2_(λ) is

$$\frac{A_{1}(\lambda )}{A_{2}(\lambda )}=\frac{\log_{10}T_{1}(\lambda )}{\log_{10}T_{2}(\lambda )}=\frac{d_{1}}{d_{2}}.$$

It is then assumed that if the ratio of the measured spectral absorbances is *d*_1_*/d*_2_ the photometric system is linear. This is not necessarily true. It is possible that the photometric system is such that the measured spectral internal transmittance *T*′(λ) is given by

$$T\prime (\lambda )={\left[T(\lambda )\right]}^{x}$$

where *x≠*1. In this case the measured spectral absorbance will be given by

$$\begin{array}{l} A\prime (\lambda )=-\log_{10}\;T\prime (\lambda )\\ \;=-x\;\log_{10}T(\lambda )\\ \;=xA(\lambda ) \end{array}$$

and the relation

$$\frac{A\prime_{1}(\lambda )}{A\prime_{2}(\lambda )}=\frac{d_{1}}{d_{2}}$$

will hold, despite the nonlinearity. Apart from this, the Lambert-Beer law is often not obeyed, especially if the measuring beam is not collimated so that the pathlength is not exactly equal to the thickness of the filter.

### B. Wavelength Errors

A survey of methods of checking the accuracy of the wavelength scales of spectrophotometers has been given recently by Alman and Billmeyer [9]. The best method for a spectrophotometer with a narrow waveband is the line spectra method, but for instruments with a wide waveband the linear filter method [10] is preferable. Again, however, these methods require auxiliary equipment and modifications to the spectrophotometers so it is convenient to have glass filters available with clearly defined transmittance minima that can be used as a simple check on the wavelength scale.

Two of the most commonly used materials are didymium [11] and holmium oxide glasses [12, 13]. One difficulty with these glasses is that the positions of the transmittance minima vary with both the width and the shape of the waveband transmitted by the spectrophotometer. The problem is especially bad when a minimum is not symmetrical or consists of two slightly separated individual minima. Holmium oxide is better than didymium in this respect but there is still some uncertainty if the waveband is not very narrow.

The effect of temperature on these filters is to increase or decrease the transmittances at the minima without changing the wavelengths significantly [14].

**Figure 3:**
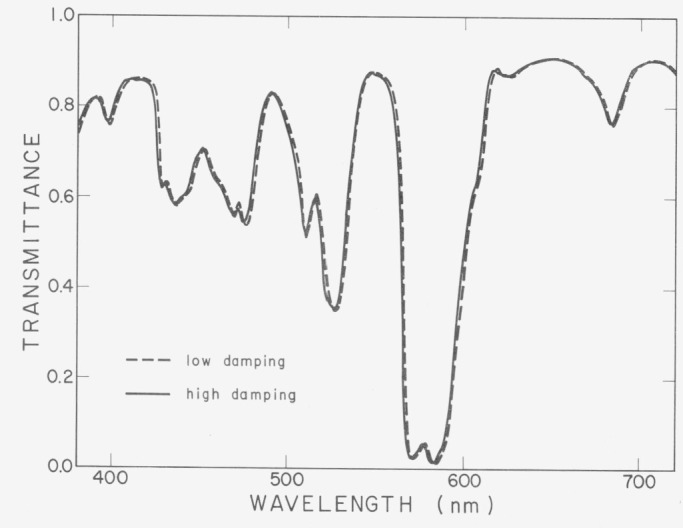
Spectral transmittance of a didymium glass measured with low and high damping of the recording system of a Zeiss DMC25 spectrophotometer. The direction of scan is from high to low wavelengths.

Both types of filter can be used with rather less uncertainty to check for changes in the performance of a single instrument, than to check one instrument relative to another. However it is sometimes difficult to distinguish wavelength errors from other types of error such as the error caused by the response of the recording mechanism being too slow to follow the changes of transmittance. An example of the latter is shown in figure 3. The broken line represents the measurement of a didymium filter measured with a fast-responding recorder and the full line represents the measurement of the same filter with a slowly responding recorder. (The direction of scan is from high to low wavelengths in both cases.)

### C. Bandwidth

**Figure 4:**
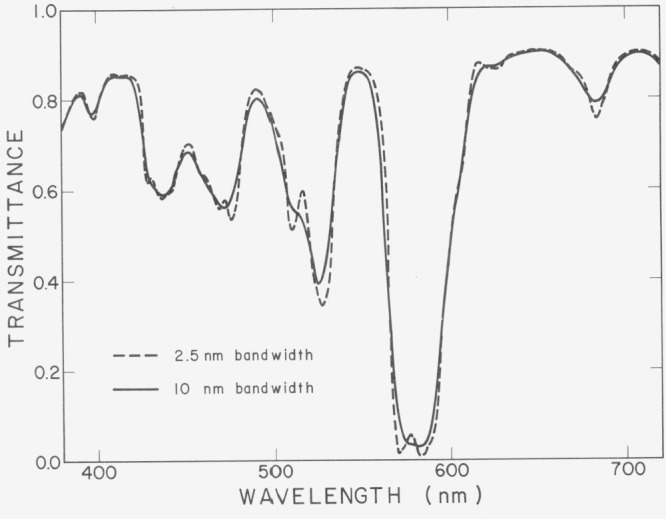
Spectral transmittance of a didymium glass measured on a Zeiss DMC25 spectrophotometer with narrow and wide slits.

No spectrophotometric measurements are ever made with radiation of a single wavelength. There is always a finite band of wavelengths distributed around the nominal wavelength. The width and shape of this band is determined by the widths of the slits of the monochromator and by the spectral power of the source, the spectral transmittance of the optics, and the spectral responsivity of the photodetector. The exact width and shape of the waveband is difficult to measure, but departures from a narrow band can be detected by measuring standards such as a didymium glass with several transmittance minima of different shapes. Figure 4 shows the spectral transmittance of such a filter measured on a Zeiss^2^ DMC 25 spectrophotometer with 2.5 nm and 10 nm bandwidths. With the wider bandwidth the minima are broadened, the wavelengths of some of the minima are shifted, and some double minima are not resolved.

It is important to check for changes of the bandwidth of a spectrophotometer, especially in those instruments, such as the Cary 14, in which the slits are adjusted automatically to maintain a constant reference signal. In such instruments, changes of the bandwidth can indicate instrumental defects such as the accumulation of dirt or the deposition of films on the optical parts.

### D. Stray Radiation

Two types of stray radiation should be distinguished. The first is stray radiation of the same wavelength as is being measured, but which reaches the detector without passing through the sample. It is caused by reflections and scattering between the various optical and mechanical components including the sample itself. It can be detected by measuring an opaque sample. If the measured spectral transmittance is not zero, stray radiation may be present, although an error of the 0 percent end-point of the photometric scale will have the same effect. Scattering or reflection by the sample can be exaggerated by painting the opaque sample white, or replacing it by a mirror.

The second type of stray radiation has wavelengths different from that of the measurement beam. It too can arise from unwanted reflections and from scattering by dirt, scratches and other defects in optical components. Another common cause is unwanted orders of diffraction in grating instruments. The effect of this stray radiation is often exaggerated greatly at the ends of the wavelength range because here the photodetector may be much more sensitive to the wavelengths of the stray radiation than to the measurement wavelength. This type of stray radiation can be detected readily by cut-off or band-pass filters that have zero transmittance for some wavelengths and high transmittance for others. Stray radiation of the former wavelengths will lower the measured transmittance at the latter, and vice-versa.

### E. Nonuniformity of Photodetector

If the response of the photodetector is not uniform across its surface, errors will occur whenever a sample causes a change in the irradiated area, either by shifting it across the photodetector or by increasing or decreasing the area by altering the focus of the measurement beam. Such errors can usually be detected by measuring clear glass or quartz filters of different thicknesses or with a wedge shape. The errors should be avoided by using a suitable diffuser to increase the uniformity of the detector response [15].

### F. Inertia Errors

**Figure 5:**
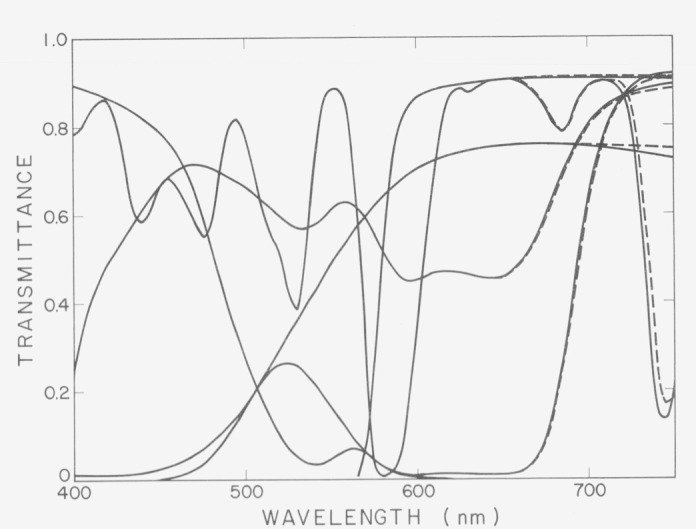
Spectral transmittances of the NBS 2100 series of filters and a didymium glass with a General Electric Hardy spectrophotometer functioning properly (full line) and with the same spectrophotometer with a malfunction causing poor response in the red part of the spectrum (broken line). The direction of scan is from low to high wavelengths.

The recording mechanism of a spectrophotometer often has significant inertia and lags or leads changes in spectral transmittance as the instrument scans through the spectrum. Mechanical or electrical malfunctions can often develop to cause such inertia even if the instrument was originally free from the problem. Errors caused by inertia can be detected readily by measuring standard meterials whose spectral transmittance varies rapidly with wavelength. An example is shown in figure 5. In this case, measurement of the NBS 2100 series of filters and a didymium glass indicated very slow response in the red region of the spectrum, but the response in the rest of the spectrum was satisfactory.

## III. Standards for the Ultraviolet

Most of the examples of diagnostic standards given in the preceding section were for the visible spectrum. Exactly the same principles apply in the ultraviolet sprectrum, but there are greater difficulties in finding suitable materials. In particular most glass filters cannot be used below about 350 nm because of their strong absorption below this wavelength. For this reason, liquid standards such as solutions of potassium chromate, copper sulphate, and cobalt ammonium sulphate have often been used [16]. Recently a number of other solutions have been suggested [17]. Unfortunately, variations in the preparing and handling of such liquid standards introduce some extra uncertainties so glass standards are preferable if they have appropriate spectral transmittance functions.

## IV. Conclusions

Material standards provide a very useful and convenient way to check the performance of a spectrophotometer and to diagnose malfunctions, although they can never completely replace more fundamental methods of checking for errors. Materials are available with some of the desired properties, especially for the visible spectral region. However most of the presently available materials do have some drawbacks. Often the spectral transmittance functions are less than ideal and in many cases they are instable to environmental conditions such as temperature and are not sufficiently permanent over long periods of time. For these reasons it is very desirable that new and better standard materials should be developed in the future.

## Footnotes

1 Figures in brackets indicate the literature references at the end of this paper.

2 In order to adequately describe materials and experimental procedures, it was occasionally necessary to identify commercial instruments. In no instance does such identification imply endorsement by the National Bureau of Standards.
